# Diseases Burden of Chronic Obstructive Pulmonary Disease (COPD) Attributable to Ground-Level Ozone in Thailand: Estimates Based on Surface Monitoring Measurements Data

**DOI:** 10.5539/gjhs.v8n1p1

**Published:** 2015-05-15

**Authors:** Chayut Pinichka, Kanitta Bundhamcharoen, Kenji Shibuya

**Affiliations:** 1 International Health Policy Program, Ministry of Public Health, Nonthaburi 11000, Thailand; 2 Department of Global Health Policy Graduate School of Medicine, The University of Tokyo, Japan

**Keywords:** ambient ozone pollution, population attributable fraction, geographic information system, spatial interpolation, burden of disease, disability adjusted life years

## Abstract

**Background:**

Ambient ozone (O_3_) pollution has increased globally since preindustrial times. At present, O_3_ is one of the major air pollution concerns in Thailand, and is associated with health impacts such as chronic obstructive pulmonary disease (COPD). The objective of our study is to estimate the burden of disease attributed to O_3_ in 2009 in Thailand based on empirical evidence.

**Methods:**

We estimated disability-adjusted life years (DALYs) attributable to O_3_ using the comparative risk assessment framework in the Global Burden of Diseases (GBD) study. We quantified the population attributable fraction (PAF), integrated from Geographic Information Systems (GIS)-based spatial interpolation, the population distribution of exposure, and the exposure-response coefficient to spatially characterize exposure to ambient O_3_ pollution on a national scale. Exposure distribution was derived from GIS-based spatial interpolation O_3_ exposure model using Pollution Control Department Thailand (PCD) surface air pollution monitor network sources. Relative risk (RR) and population attributable fraction (PAF) were determined using health impact function estimates for O_3_.

**Result:**

PAF (%) of COPD attributable to O_3_ were determined by region: at approximately, Northern = 2.1, Northeastern = 7.1, Central = 9.6, Eastern = 1.75, Western = 1.47 and Southern = 1.74. The total COPD burden attributable to O_3_ for Thailand in 2009 was 61,577 DALYs. Approximately 0.6% of the total DALYs in Thailand is male: 48,480 DALYs; and female: 13,097 DALYs.

**Conclusion:**

This study provides the first empirical evidence on the health burden (DALYs) attributable to O_3_ pollution in Thailand. Varying across regions, the disease burden attributable to O_3_ was 0.6% of the total national burden in 2009. Better empirical data on local specific sites, e.g. urban and rural areas, alternative exposure assessment, e.g. land use regression (LUR), and a local concentration-response coefficient are required for future studies in Thailand.

## 1. Introduction

Ground-level ozone or ambient ozone pollution (O_3_) is one of the major air pollution concerns at both national and global levels, which are associated with health impacts, such as premature mortality due to respiratory infection (Huang, Dominici, & Bell, 2005; Ito, Thurston, & Silverman, 2007; M. Jerrett et al., 2009). The Global Burden of Diseases study 2010 (GBD, 2010) (WHO, 2011) estimated that the burden attributed to O_3_ exposure distributions accounted for 0.2 million or 0.1% of global DALYs in 2010, approximately 6.3% larger than the burden in 1990 (Lim et al., 2012). A study by Anenberg et al (Anenberg, Horowitz, Tong, & West, 2010) using the chemistry transport model, estimated global annual respiratory mortality of 0.7 ± 0.3 million (6.3 ± 3.0 million years of life lost), or 1.1% ± 0.5% of all respiratory mortalities were associated with O_3_ pollution. In addition, almost 80% of the total global O_3_ pollution impact in this study occurred in Asia. Since 1997, O_3_ has dramatically exceeded the standard level in many areas of Thailand because of rapidly expanding cities, increasing population density, increasing trend in the number of fossil fuel vehicles and electricity generation (Ruchirawat, Settachan, Navasumrit, Tuntawiroon, & Autrup, 2007; Thanh & Lefevre, 2000). O_3_ can be formed in mega cities and carried toward rural areas and distributed across the country via atmospheric transport pathways, therefore, it is necessary to quantify the health burden of O_3_ pollution at the national level.

Global estimates of ozone by the powerful chemistry transport model may be under or over-estimating the results because it does not use national level empirical data at the local level. Thailand has used O_3_ surface monitored measurements from the Pollution Control Department in recent years, which when used with data from geographic information system (GIS), can improve simulated O_3_ distribution at a specific level. GIS is a well-known program and has been used to estimate the exposure distribution in many environmental epidemiology studies including O_3_ pollution exposure (Moral García et al., 2010; Hunova, 2011; Nuckols & Lars Jarup, 2004; Verónica, 2013). To estimate the health burden attributed to O_3_ in Thailand, it is necessary to develop an accurate prediction of the distribution of O_3_ exposure values at non-measurement locations with the empirical data available at the local level.

Our main objective was to estimate the attributable burden of disease due to O_3_ exposure in Thailand using the spatial interpolation of O_3_ concentrations, health impact function and calculated disability-adjusted life years (DALYs), which are a composite metric that measures both deaths and disabilities, combined with the comparative risk assessment method (Lim et al., 2012; Murray, 1994).

## 2. Method

### 2.1 Overall Approach to Estimating Burden Attributable to O_3_

GBD uses the disability-adjusted life-year (DALY) as developed by the Burden of Disease workgroup at the World Health Organization (WHO) to quantify the burden of disease. DALY is the sum of the years of life lived with a disability (YLD) and years of life lost (YLL). Our methods are based on the GBD 2010 comparative risk assessment (Lim et al., 2012). We use an integrated method that combines exposure assessment based on surface monitoring measurements and comparative risk assessment (CRA) to quantify the burden of disease attributed to ambient O_3_ pollution as shown in Table 1. The comparative risk assessment method used to calculate the attributable burden due to O_3_ in this study is based on epidemiological evidence for O_3_ exposure. Exposure-response relationships for O_3_ are derived from epidemiological studies in GBD 2010, to calculate attributable fractions, and are then multiplied by disease burden, and expressed in DALYs attributable to O_3_ (WHO, 2004).

**Table 1:** Risk factors included, exposure variables, theoretical-minimum-risk exposure distributions, and outcomes affected

Air pollution type	Exposure definition	Outcomes	Subgroup	Main data sources for exposure	Exposure estimation method	Theoretical minimum-risk exposure distribution	Source of relative risks
**Ambient ozone pollution**	Ambient concentrations of ozone in air, measured in parts per billion	COPD	Age ≥25 years	Surface monitor measure-ments	Surface monitor measurements and GIS interpolation	33.3–41.9 parts per billion	Jerrett and Colleague (M. Jerrett et al., 2009)

Essentially, the estimation of the burden attributable to O_3_ consists of three main steps: (1) measuring the total burden of disease associated with the risk factor at the population level, (2) estimating the population attributable fraction (PAF), and finally (3) applying the PAF to the total burden of disease.

### 2.2 Data Sources

#### 2.2.1 O_3_ Exposure

**Figure 1:**
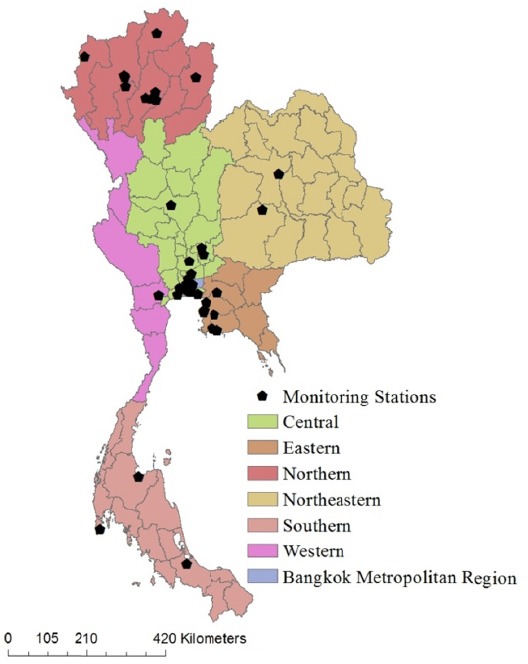
Area of the study and location of surface monitoring stations network

The most important part of the O_3_ concentration estimation is the extrapolation of pollutant levels at different spatial locations based on empirical data from air quality monitoring stations in Thailand. The monitoring stations are located in six-regions (Northern, Northeastern, Eastern, Western, Central, and Southern) and monitor particulate matter (PM 10), fine particles (PM 2.5), CO, NO_2_, SO_2_, and ground-level ozone. The stations are operated by the Thailand Pollution Control Department (PCD), which has been monitoring O_3_ since 1992 through the Thailand air quality monitoring network. The network consists of 55 continuous monitoring stations in 23 provinces, and 52 O_3_ monitor stations across the country (Pollution Control Department, 2007). Figure 1 illustrates the location of these air quality monitoring stations and regions, and Table 2 shows the number of O_3_ surface-monitoring stations by regions in Thailand.

**Table 2:** Number of O_3_ surface monitor stations and regional categorization in Thailand

Locations	Number of stations
**Central (including Bangkok Metropolitan Area)**	28
**Northern**	10
**Northeastern**	2
**Eastern Western**	8 1
**Southern**	3

**Total**	52

*Note.* source: (Pollution Control Department, 2007).

Geo-statistics and spatial exposure modeling techniques were applied to air pollution exposure assessment in a number of environmental-health studies (Hunova, 2011; M. Jerrett et al., 2013; Thepanondh & Toruksa, 2011). The spatial modeling technique estimates the unknown value of an un-observed area within the area covered by using an existing known value observation. GIS allowed us to study and analyze spatial distributions of O_3_ pollution on a national level based on empirical data. Because of these advantages, GIS can also be a powerful tool to show spatial distribution of the exposure. Without a GIS assessment, exposure distribution analysis of national air pollution data would be difficult to obtain.

Inverse distance weighting (IDW) is a deterministic interpolation method and based on the nonlinear interpolation technique that uses a weighted average of the attribute values from nearby known value data to estimate the value of that attribute at unknown data locations. Moreover, IDW showed good results for assessment and monitoring of ambient air quality parameters (Dilip Kumar Jha, Sabesan, Anup Das, Vinithkumar, & Kirubagaran, 2011).

The model formula of IDW interpolation is given by;


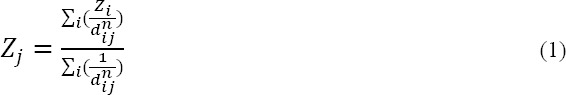


Z_j_ is the estimated of O_3_ concentrations value at location j;

Z_i_ is the measured of O_3_ concentrations value at location i;

d_ij_ is the distance from measured value at location i to the estimated value at location j;

n is the number of O_3_ concentrations measured value points used for interpolation.

In addition to the IDW method, several studies used the Kriging method to evaluate O_3_ exposure (Denby et al., 2010; Gorai, Tuluri, & Tchounwou, 2014; Kethireddy, Tchounwou, Ahmad, Yerramilli, & Young, 2014; Roberts, Voss, & Knight, 2014). Kriging weights the distance between measured locations based on its spatial correlation to produce variograms and a covariance factor for predicting the unknown locations (Wong, Yuan, & Perlin, 2004). In this study, cross-validation analysis was used to evaluate the performance of the spatial interpolation techniques and the uncertainty of the maps (Janssen, Dumont, Fierens, & Mensink, 2008); we used Pearson correlation (r) between the measured values at the monitoring stations and the model predictions. We assessed the model performance by statistical performance indicators comparing the Root Mean Squared Error (RMSE) and Mean Absolute Error (MAE). The equation is given by;


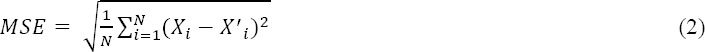



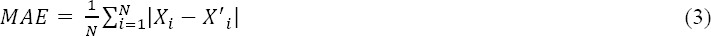


Where, X_i_, X’_i_ and N are measured, estimated and number of variables.

#### 2.2.2 Relative Risks and Population Attributable Fraction

The population attributable fraction (PAF) has long been used to estimate the proportion reduction of burden that can be attributed to specified risk factors (Levin, 1953; Rockhill, Newman, & Weinberg, 1998). If these risk factors were eliminated, it can be concluded that the burden would be reduced from these risk factors. Generally, an exposure-based approach to determine the PAF attributed to O_3_ requires three components of data: the exposure of the O_3_, the population of exposure (Pe), and the exposure-response relationships or Relative Risk (RR). We calculated the health impact function for O_3_ based on a log-linear relationship between relative risk (RR) and concentrations defined by an epidemiological study (M. Jerrett et al., 2009). We used relative risk from this study because it is the first study that showed significant O_3_ long-term health impact. In addition, the GBD 2010 only used RR from this study for their estimations. We assumed that the background O_3_ concentrations, the relationship between O_3_ and health impact in Thailand, are on the same scale as the GBD 2010. The health impact function was evaluated based on the relationship between relative risk (RR) and the change in O_3_ concentrations, defined as follows;


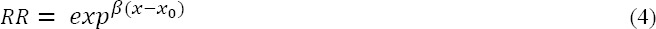


Where β is the concentration-response coefficient (CR), which is the slope of the log-linear between O_3_ concentrations and mortality, and x - x_0_ or Δx is the concentration change from baseline conditions (x_0_), defined as follows:

X = Average annual 1-hours daily maximum O_3_ concentrations in 1997-2009 (ppb)

X_0_ = Theoretical minimum or background concentrations (ppb)

We calculated the burden of disease attributable to ambient O_3_ pollution by multiplying the total disease burden and the PAF which was determined by equation 5 (Murray, Ezzati, Lopez, Rodgers, & Vander Hoorn, 2003), where Pe is the population distribution of the exposure and RR is the relative risk.


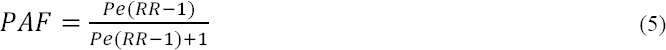


The exposed population may itself be divided into multiple categories based on level or length of exposure each with its own relative risk. With multiple (n) exposure categories, the PAF is given by the following generalized form(Murray et al., 2003):


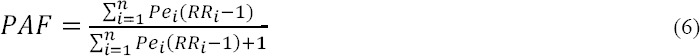


PAF=PAF = Proportion of disease burden attributable to O_3_.

Pe_i_ = Proportion estimates of the population that’s exposed to O_3_ by grid i.

RR_i_ = Relative risk (magnitude of the association between O_3_ and disease) by grid i.

**Figure 2:**
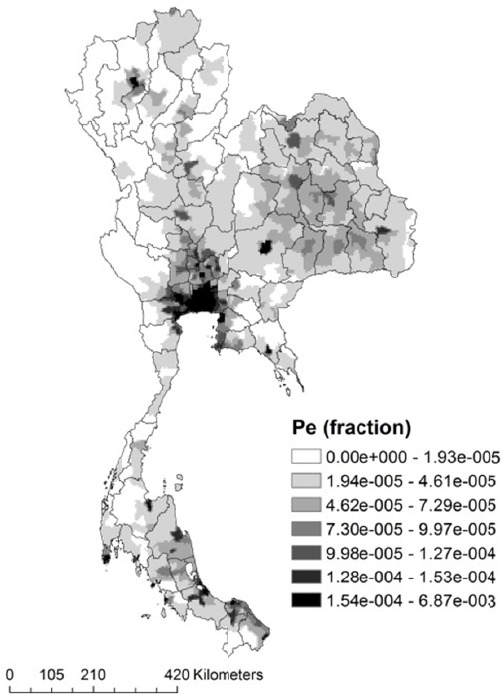
Geographic population distribution of exposure (Pe)

We used relative risk of chronic obstructive pulmonary disease (COPD) from Jerrett et al. (M. Jerrett et al., 2009). The population data used in this study was obtained from the 2000 Gridded Population of the World, Version 3 (GPWv3) generated by the SEDAC (Socioeconomic Data and Applications Center) project at Columbia University (CIESIN, 2005). This population dataset estimated human population from national and subnational input sources (usually administrative sources) of varying resolutions into regular latitude- longitude grids at the resolution of 2.5 arc-minute grid cells (or ~5km at the equator). All population ages ≥ 25 years is assumed to be exposed because it is not known exactly what proportion of the population is exposed to air pollution at the national level (AIHW, 2010). We used Pe as the proportion of population at ages ≥ 25 years, which was obtained from Department of Provincial Administration Thailand (Department of Provincial Administration, 2009), and assumed to be constant across the country (Figure 2).

#### 2.2.3 Estimating Burden Attributable to O_3_

To estimate the disease burden attributable to O_3_, the attributable burden (AB) is multiplied by the total COPD disease burden (TB) estimates for the disease and the attributable fraction (PAF). We then estimated the disease burden attributable to O_3_ according to the formula:


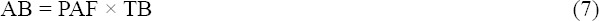


AB = PAF × TB (7)

PAF = Proportion of cases that attributed to O_3_.

TB = Total COPD disease burden (DALYs).

The total COPD disease burden (DALY) dataset was obtained from BOD Thailand (Thai BOD) (BOD, 2009), which published the national disease burden in Thailand (BOD, 2009). We assumed that the ratio of males to females (sex ratio), is on the same scale as the Thai BOD (Bundhamcharoen, Odton, Phulkerd, & Tangcharoensathien, 2011).

## 3. Results

**Figure 3:**
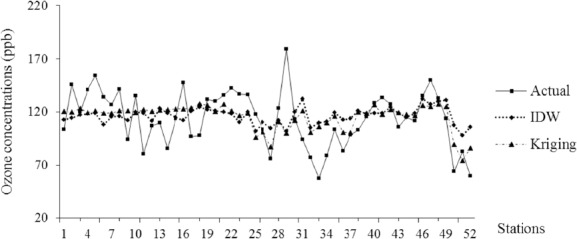
Predicted and actual average daily maximum O_3_ concentrations (1997-2009) by monitoring stations

**Figure 4:**
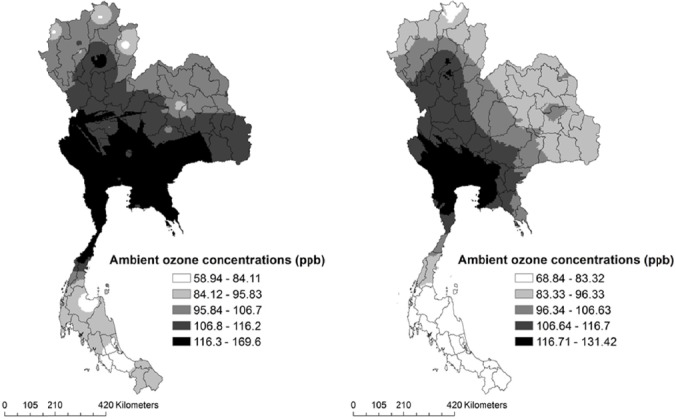
Exposure maps of O_3_ concentrations in Thailand (2009), IDW (left) and Kriging (right)

We present a number of comparisons of the results using the different spatial modeling described in section 2. The statistical summary of model performance corresponding to Kriging and IDW interpolation methods are presented in Table 3. Considering the average from the O_3_ concentrations of 1997 and 2010, the mean predicted O_3_ concentration of IDW and Kriging in Thailand were: 108.25 ppb (95% CI, 108.1-108.4) and 99.7 ppb (95% CI, 99.5-99.8). In this case, IDW was shown to have an approximately 8.6% larger mean predicted concentration than the Kriging model. Both interpolation models showed that the average O_3_ concentrations in Thailand exceeded the standard. Furthermore, the IDW model showed the maximum O_3_ concentration to be approximately 169.6 ppb, while the Kriging model gave 131.4 ppb, or a 29% difference. Figure 3 illustrates the cross validation between the measured values at the monitoring stations and the model predictions. For both models, the Pearson correlation of the IDW model (r = 0.272) was lower than the Kriging model (r = 0.5). In addition, the model performance assessments showed that the MAE and RMSE for O_3_ varied from 17.9 to 20.25 and 22.58 to 25.13, respectively. These results revealed that the Kriging interpolation method performed with 10-12% better accuracy than the IDW method, and clearly indicates that the Kriging provides optimal interpolation model for the exposure assessment based on the empirical dataset in the present study. In addition, the spatial distribution of O_3_ concentration from the IDW and Kriging model are presented in Figure 4. The Kriging model clearly indicates that the highest O_3_ concentration levels in the Central and Eastern regions with a range of 117-131 ppb and, the lowest levels in southern region, with a range of 69-83 ppb.

**Table 3:** Statistical summary and model performance parameters of spatial interpolation model

Statistics measured	Interpolation methods (unit : ppb)	

	IDW	Kriging
**Mean**	108.25	99.7
**95% Confidence Interval for Mean**	108.1-108.4	99.5-99.8
**Median**	109.42	98.42
**Std. Deviation**	12.03	14.17
**Minimum**	58.94	68.84
**Maximum**	169.62	131.42
**Correlation coefficients (*P*-value))**	0.272 (0.051)	0.50* (0.00)
**MAE**	20.25	17.9
**RMSE**	25.13	22.58

* *Note.* Correlation is significant at the 0.01 level (2-tailed).

We then calculated ΔX in six regions and discovered that the average ΔX values were between 64.7-84.9 ppb in Thailand (Table 4). We calculated the summation of PAF grids (Figure 4) by region, as shown in Table 4. Our best PAF estimation indicates that the western region had the lowest PAF value, or about 1.47% of the total COPD burden. The highest PAF value was located in the central region, which produced 9.6% of the total COPD burden reflecting the highest O_3_ concentration levels.

**Table 4:** Population ≥ 25 years, change in concentrations, relative risk and population attributable fractions

Region	Pop ≥ 25 (millions)	Δx		RR		PAF grid_i_ (% of total COPD DALYs)	

		Mean	95% CI for mean	Mean	95% CI for mean	Mean	95% CI for mean
**Northern**	3.9	64.7	57.9-71.4	1.29	1.26-1.32	0.0007	0.0006-0.0008
**Northeastern**	13.7	60.8	58.8-62.7	1.27	1.26-1.28	0.0014	0.0013-0.00143
**Central**	12.7	84.9	81.8-88.04	1.39	1.38-1.41	0.005	0.004-0.006
**Eastern**	2.4	80.5	74-86.9	1.37	1.34-1.40	0.0014	0.0013-0.0015
**Western**	1.9	83.97	76.7-91.2	1.39	1.35-1.43	0.0008	0.0007-0.0009
**Southern**	5.2	43.5	40.4-46.6	1.19	1.17-1.20	0.00058	0.0005-0.0006

**Figure 5:**
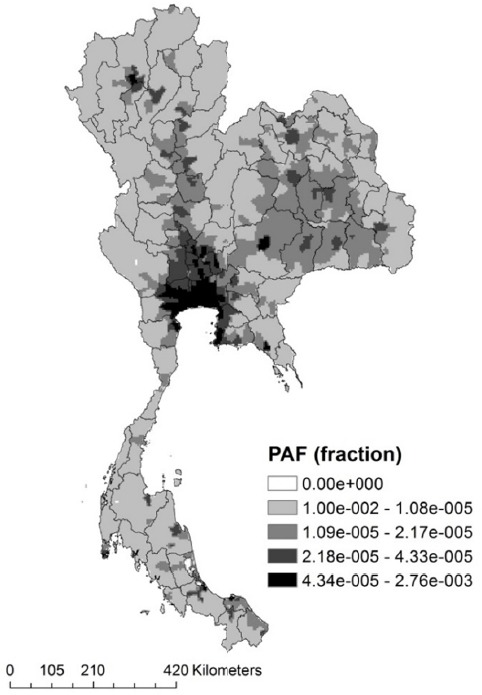
Spatial variation of population attributable fractions (PAF) to COPD in Thailand (2009)

Table 5 summarizes the entire attributable burden due to O_3_ pollution by region in Thailand. The total burden of disease in Thailand was 10.2 million DALYs, and the estimated total COPD burden in Thailand was 259,512 DALYs (male: 204,312 DALYs; female: 55,199 DALYs) (BOD, 2009). Figure 5 illustrates the distribution of population attributable fractions (PAF) to COPD in Thailand. The Total COPD burden attributable to O_3_ among six regions was approximately 61,577 DALYs (0.6% of total DALYs) (male: 48,480 DALYs; female: 13,097 DALYs). The highest attributed O_3_ burden was clearly located in the central region including the Bangkok Metropolitan Area. The burdens were approximately 9.6% of the total COPD burden, or about 24,812 DALYs, while the lowest attributed burden was located in the western region, which approximately 1.47% of total COPD burden, or about 3,804 DALYs.

**Table 5:** Attributable O_3_ burden in Thailand

Region	Attributable O_3_ burden (DALY)			Total DALYs (millions)	% of total DALYs

	Both sexes	Male	Female		
**Northern**	5,490	4,322	1,168	1.4	0.39
**Northeastern**	18,430	14,510	3,920	3.3	0.55
**Central**	24,813	19,535	5,278	3.0	0.83
**Eastern**	4,531	3,567	963	0.7	0.69
**Western**	3,804	2,995	809	0.5	0.77
**Southern**	4,510	3,551	959	1.3	0.35
**Total**	61,577	48,480	13,097	10.2	0.60

## 4. Discussion

This study estimated the health burden of diseases due to O_3_, using GIS interpolation models and health impact function, and followed the comparative risk assessment method (CRA) from the global burden of disease (GBD) study. This approach makes it possible to assess the burden attributable to empirical data sources for O_3_ exposure, e.g., surface monitor measurements, while taking into consideration the population exposure at the national level. Our results indicate that the burden of disease attributable to O_3_ is approximately 61,577 DALYs (0.6% of total DALYs) (male: 48,480 DALYs; female: 13,097 DALYs). The only source of relative risks due to O_3_ from the GBD study is Jerrett et al. (2009), a major cohort study in the United States and also the first study to show significant O_3_ exposure long-term health impacts.

We estimated O_3_ exposure by two spatial exposure-modeling techniques, i.e., IDW and Kriging. The cross-validation technique was used to test the model accuracy from the air pollution exposure study (Thepanondh & Toruksa, 2011), because it does not require additional data collection from areas without an air surface monitoring station. It removes a known point from the interpolated dataset and the residual dataset is used to evaluate the prediction at the known point that it had been removed. The model performance indicator of each of the interpolation methods is shown in Table 4, and the predicted cross-validation data is shown in Figure 2. Both interpolation techniques were useful for estimating the exposure from the unknown areas, but the results showed that the Kriging, which produced smaller RMSE and MAE values, performed better than IDW. Using the Kriging method as shown in Figure 4, an O_3_ concentration exposure map from spatial distributed interpolation was produced.

From our study, the average ΔX value in Thailand = 69.3 ppb (95% CI 62-70). These figures are larger than Horowitz’s simulation at 10-25 ppb (Horowitz, 2006); Horowitz used the chemistry transport model to generate globally simulated O_3_ concentrations of 30-50 ppb in area of Thailand. To improve these results at the national level, increasing the number and distribution of monitoring stations is required in the future because of a lack of representative surface monitored data in some high population provinces (populations > 1 million) in Thailand. For example, Ubon ratchathani, Udon Thani and Buriram in northeastern region, Nakhon Si Thammarat in southern region, all high population provinces, lack representative surface monitored data.

Figure 4 illustrates how the population attributable fraction due to O_3_ exposure varies across Thailand. The population attributable fraction was the highest in the Central region (including Bangkok Metropolitan Area), most likely because the higher population distribution of exposure (Figure 2) and O_3_ concentrations levels (Figure 4) are located in this region. Furthermore, we found that the spatial variation of population attributable fractions were generally more consistent with the population distribution of exposure than the O_3_ concentrations, similar to the previous study that suggested the exposure comes with population; no population, no exposure (Hao, Flowers, Monti, & Qualters, 2012). The highest attributable COPD burden due to O_3_ was located in Central region, or about 24,812 DALYs. This may be due to the population distribution of exposure and pollution levels, but also because the total COPD DALY in the Central region was consistently high as well. However, the Northern region, which had the highest COPD DALY in Thailand, had lower estimated O_3_ attributable disease burden than the Central region (5,490 DALYs). This may be because the O_3_ concentrations were slightly lower (64.7 ppb, 95% CI; 57.9-71.4) than the Central region (84.9 ppb, 95% CI; 81.8-88.04), the population distribution of exposure was relatively smaller than the Central region, and the major sources of air pollution which are associated with COPD in the Northern region may differ from the Central region (e.g. forest fire, and garbage burnings)(Sukitpaneenit & Kim Oanh, 2014; Wiwatanadate, 2014).

In a previous study, GBD 2010 (Lim et al., 2012), estimated the disease burden attributable to O_3_ to be approximately 0.1% of the global DALYs (152,434 deaths: both sex) in 2010, our best estimate, however, is 0.45-1%, depending on the province/region, or a total of 61,577 DALYs (0.6% of the total national disease burden). Our results indicate that our PAFs were slightly greater than the GBD study. One explanation may be that the exposure estimation method. GBD used the global chemistry transport models (Fiore, Dentener, Wild, Cuvelier, & Schultz, 2009; Stevenson et al., 2006) to estimate the distribution of O_3_. GBD did not link the available surface monitor data and statistical model to estimate exposure distribution similar to the fine particulate matter (PM2.5) in their study, which reflects a lack of available local empirical data. In addition, the estimations for the O_3_ disease burden in Thailand are approximate to the United Arab Emirates (UAE). Based on the air quality monitoring stations in the UAE, and the spatial exposure modeling technique, Ying Li et al. (Li et al., 2010) estimated that disease attributed to ground-level O_3_ exposure in UAE was approximately 0.8% (95% CI; 0.2–2%) of the total UAE disease burden in 2007.

We note that our study has some inherent limitations, which need to be addressed. We rely on O_3_ pollution data from existing monitoring stations network. In addition, the exposure assessment based on the surface monitoring measurements may vary, depending on location, air quality monitoring stations density and seasonal variation in O_3_ pollution which may not be uniformly distributed across Thailand. For that reason, it is possible that our model was overestimates to simulate annual O_3_ exposure into large scale area (e.g. national scale). We recommend that future research should consider another ambient air quality model to simulate O_3_ concentrations, e.g. land use regression (Ryan & LeMasters, 2007) and photochemical modeling (Fann et al., 2012) to more accurately assess O_3_ pollution and population exposure on a national scale.

In the study limitation, we assumed that the exposure was equal for all members of the population ≥ 25 years (AIHW, 2010). For that reason, it should be noted that using an urban/local area-weighting method to estimate population exposure (Zhang, Qi, Jiang, Zhou, & Wang, 2013) due to O_3_, and the higher resolution population dataset at a resolution of 30 arc-seconds (~1km), which allocating census block population and household information into regular latitude longitude grids (Balk et al., 2006), are required to improve the contribution on the Pe factor at the local level.

Another limitation is that our study relies on the health outcome model from the GBD study (Lim et al., 2012), and also concentration–response coefficients (CR) from an epidemiological study (M. Jerrett et al., 2009) despite differences in health status, lifestyle, age structure, and medical care (Anenberg et al., 2010). Therefore, we did not account for short-term effects by the ambient O_3_ pollution, which is related to cardiovascular and respiratory mortality (e.g. Bell et al., 2004). These limitations may be eliminated in future research if local epidemiological studies are available.

## 5. Conclusion

Our study aims to estimate the disease burden attributed to ozone in Thailand. This study presents an integrated exposure assessment, using a spatial interpolation model from empirical data, population distribution exposure and health impact function to estimate national disease burden attributable to O_3_. Our study indicated that ambient O_3_ is one of the major air pollutants that exerts adverse effects on the environment and human health in Thailand. The estimated COPD burden cause by O_3_ in our study was about 0.6% of the national burden each year. It is our hope that the results will initiate more precise O_3_ and/or other air pollution health burden estimations among scientists in future environmental health burden studies. Finally, this study provides the first national estimate and can inform decision-making by stakeholders and policy-makers to promote and manage a health co-benefit of green economy in the future.
